# Acid suppressants use and the risk of dementia: A population-based propensity score-matched cohort study

**DOI:** 10.1371/journal.pone.0242975

**Published:** 2020-11-30

**Authors:** Chia-Liang Wu, Wei-Yi Lei, Jaw-Shing Wang, Ching-En Lin, Chien-Lin Chen, Shu-Hui Wen

**Affiliations:** 1 Department of Psychiatry, Taipei Veterans General Hospital, Hualien City, Taiwan; 2 Institute of Medical Sciences, Tzu Chi University, Hualien City, Taiwan; 3 Department of Internal Medicine, Buddhist Tzu Chi General Hospital, Hualien City, Taiwan; 4 Department of Psychiatry, Taipei Tzu Chi Hospital, New Taipei City, Taiwan; 5 School of Medicine, Tzu Chi University, Hualien City, Taiwan; 6 Department of Public Health, College of Medicine, Tzu Chi University, Hualien City, Taiwan; National Yang-Ming University, TAIWAN

## Abstract

In this population-based propensity score matched (PSM) cohort study, we aimed to investigate the risk of developing dementia with the use of acid suppressants, including proton pump inhibitors (PPIs) and histamine-2 receptor antagonists (H2 antagonists). Cohorts of PPI users (n = 2,778), H2 antagonist users (n = 6,165), and non-users (n = 86,238) were selected from a dataset covering the years 2000 to 2010 in Taiwan’s National Health Insurance Research Database. Patients in the three groups were PSM at a ratio of 1:1 within each comparison cohort (CC). Three CCs were created: (1) PPI users compared to non-users (CC1, n = 2,583 pairs); (2) H2 antagonist users compared to non-users (CC2, n = 5,955 pairs); and (3) PPI users compared to H2 antagonist users (CC3, n = 2,765 pairs). A multivariable robust Cox proportional hazard model was used to estimate the adjusted hazard ratio (aHR) and the 95% confidence interval (CI) for the risk of developing dementia. The multivariable analysis results show that the aHR of developing dementia during the follow-up period was 0.72 (CC1: 95% CI = 0.51–1.03, *P* = 0.07) for PPI users and 0.95 (CC2: 95% CI = 0.74–1.22, *P* = 0.69) for H2 antagonist users, when compared to non-users. Between the patients using acid suppressants, there was no difference between PPI and H2 antagonist users in the risk of developing dementia (CC3: aHR = 0.82, 95% CI = 0.58–1.17, *P* = 0.28). In conclusion, no association was observed between the use of acid suppressants and the risk of developing dementia in any of the three CCs. Further, randomized controlled trials are warranted to confirm this relationship.

## Introduction

Peptic ulcer and gastroesophageal reflux disease (GERD) are common acid-related gastrointestinal diseases. The global prevalence rates of peptic ulcer and GERD have been estimated to be between 5–10% in the general population [1] and 10–20% in Western countries [2]. The primary available treatment is gastric acid suppressants, such as proton pump inhibitors (PPIs) and histamine-2 receptor antagonists (H2 antagonists) [3, 4], whereby the mechanisms underlaying the action of these acid suppressants differ [4]. Previous *in vitro* and *in vivo* studies have reported a potential link between acid suppressants and the decline of cognitive function [5–7]. Therefore, it is essential to investigate whether the use of these drugs in clinical settings is associated with the risk of developing dementia.

Currently, the associations between acid suppressants and the risk of developing dementia remain controversial. The increased risk of dementia development in PPI users has been reported in longitudinal studies [8–11] and a recent systematic review paper [12]. Conversely, other studies found a decreased risk [13] or no association [14–18] between PPIs use and dementia development. In H2 antagonist users, previous studies examining the risk of developing dementia also yielded inconsistent findings [17, 19, 20]. In addition, one study found that the risk of dementia development in PPI users was similar to that in H2 antagonist users [21]. Hence, the risk of developing dementia due to acid suppressants use remains unclear. Notably, these studies compared the risk of developing dementia between two groups such as PPI or H2 antagonist versus non-users, or PPI versus H2 antagonists. To investigate the overall associations regarding this issue, it is necessary to perform a complete study, involving risk comparisons of dementia development among all three groups from the same population, namely the PPI, H2 antagonist, and non-user groups.

Herein, we investigated the risk of developing dementia in relation to the use of acid suppressants, aiming to add more evidence to this topic. To do that, we created and studied three propensity-score matching (PSM) comparison cohorts (CCs), CC1: PPI users compared to a non-users group, CC2: H2 antagonist users compared to a non-users group, and CC3: PPI users compared to H2 antagonist users group, using Taiwan’s National Health Insurance Research Database (NHIRD).

## Materials and methods

### Data source

The Taiwanese universal National Health Insurance (NHI) program, established in 1995, is a single-payer compulsory social insurance plan. It covers all types of healthcare institutions and is implemented on approximately 99% of the population. The NHI database contains registry files and original claims of inpatients and ambulatory patients. The NHIRD was jointly generated by the National Health Insurance Administration (formerly the Bureau of NHI) and the Ministry of Health and Welfare (formerly the Department of Health). The NHIRD is maintained by the National Health Research Institutes and is openly accessible to scientists in Taiwan for research purposes. This study used the Longitudinal Health Insurance Database 2000 (LHID 2000), a cohort dataset comprised of 1,000,000 beneficiaries, randomly sampled from all beneficiaries in Taiwan in 2000. There is no significant difference in the distribution of age and sex between individuals in the LHID 2000 and the entire beneficiary population [22]. We used cases listed in LHID 2000, dating between January 1^st^, 2000 and December 31^st^, 2010. The patients’ identification numbers in the LHID 2000 have been encrypted to protect their privacy. Dispensed drug information was extracted based on the records of hospitals, clinics, and contracted pharmacies. Information on disease diagnosis was extracted from ambulatory visit records, following the International Classification of Diseases, Ninth Revision, Clinical Modification (ICD-9-CM). This study was exempt from a full review by the Institutional Review Board (IRB) of the Buddhist Tzu Chi General Hospital, Hualien, and informed consent was waived (IRB number: 106-96-C). Our research was performed following relevant guidelines/regulations.

### Study design and patient selection

**Fig 1:**
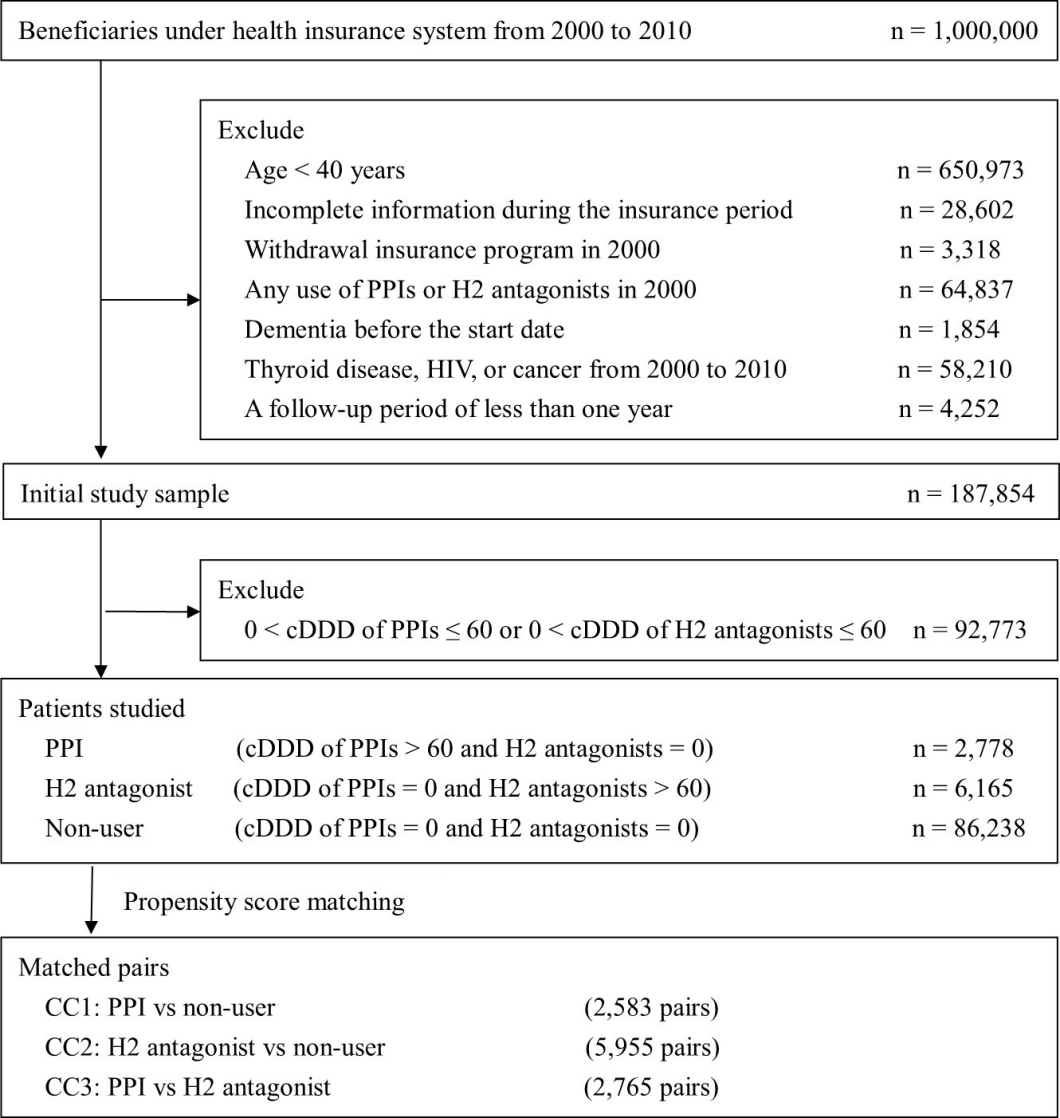
Flowchart of study sample selection. CC, comparison cohort; cDDD, cumulative defined daily dose; H2 antagonist, histamine-2 receptor antagonist; HIV, human immunodeficiency virus; PPI, proton pump inhibitor.

A retrospective population-based cohort study was designed to compare the risk of developing dementia in three PSM CCs, including (1) PPI users compared to non-users group, (2) H2 antagonist users compared to non-users group, and (3) PPI users compared to H2 antagonist users group. Exclusion criteria included: (1) Age < 40 years, (2) incomplete information during the insurance period or withdrawal from the insurance program in 2000, (3) any use of PPIs or H2 antagonists in 2000, (4) dementia (ICD-9-CM code: 290.xx, 294.1, 331.0) prior to the start date, (5) thyroid disease (ICD-9-CM code: 240.xx-246.xx), human immunodeficiency virus (ICD-9-CM code: 042), or cancer (ICD-9-CM code: 140.xx-239.xx) from 2000 to 2010, and (6) a follow-up period of less than one year. The initial study sample included non-users of acid suppressants and new users of either PPIs or H2 antagonists were those recorded in the database between 2001 and 2010. The drug exposure measure for PPIs and H2 antagonists was a cumulative defined daily dose (cDDD). Defined daily dose (DDD), a statistical tool for presenting drug utilization, was used to standardize the exposure to PPIs or H2 antagonists in different classes of medications. All PPIs and H2 antagonists were identified based on the DDD, as defined by the World Health Organization [23]. cDDD was subsequently calculated as the sum of DDDs of drug use from the first prescribed date of medication to the end of the follow-up. PPI and H2 antagonist prescriptions were identified in ambulatory visits and contracted pharmacies during the follow-up period, using the anatomical therapeutic chemical code (ATC code) [23] for classification (S1 Table). The PPI group was defined as individuals treated with PPIs > 60 cDDD and without using H2 antagonists during the study period (between 2001 and the end date). The H2 antagonist group consisted of individuals treated with H2 antagonists > 60 cDDD and without using PPIs during the study period. The non-user group was defined as individuals without any use of PPIs or H2 antagonists. There were 2,778, 6,165, and 86,238 subjects in the PPI, H2 antagonist, and non-user groups, respectively (Fig 1).

We also applied PSM at a ratio of 1:1 for each CC to reduce bias and balance confounding factors at baseline between the group pairs. That is to say, CC1 of PPI users versus non-users, CC2 of H2 antagonist users versus non-users, and CC3 of PPI users versus H2 antagonist users. Propensity score (PS) for each CC, i.e., the predicted probability of either PPI use (for CC1 and CC3) or H2 antagonist use (for CC2), was separately calculated using logistic regression based on baseline demographics (age and sex), the year of the start date, and comorbidities. The baseline of this study was defined as one year before the start date of each subject. Baseline comorbidities included cardiovascular diseases (ICD-9-CM code: 410.xx–414.xx), diabetes mellitus (ICD-9-CM code: 249.xx, 250.xx), hyperlipidemia (ICD-9-CM code: 272.xx), hypertension (ICD-9-CM: 401.x–404.x), stroke (ICD-9-CM code: 430.xx, 431.xx, 432.xx, 433.xx, 434.xx, 436.xx, 437.1), and chronic renal failure (ICD-9-CM code: 585.xx). Based on previous studies [24–26], gout (ICD-9-CM code: 274.xx), and asthma (ICD-9-CM code: 493) have also been identified as baseline comorbidities. These comorbidities were identified following the entries of least two ambulatory visits within one year before the start date. The comparator group was matched based on the logit function on PS within calipers, with a standard deviation of 0.2 of the logit of the PS [27, 28]. To this end, the total sample size of PSM pairs for each CC was as follows: 2,583 PSM pairs in CC1, 5,955 PSM pairs in CC2, and 2,765 PSM pairs in CC3 (Fig 1). The start date of follow-up was defined as the date of cDDD of PPIs > 60 for the PPI group and the date of cDDD of H2 antagonists > 60 for H2 antagonist groups, respectively. For CC1 and CC2, the start date for the non-user group was defined as the same as that for the matched acid suppressants users.

### Outcome measure

The study outcome was dementia. To identify dementia accurately, patients were determined to have the primary diagnosis of dementia following at least two ambulatory visits during the follow-up period. In Taiwan, psychiatrists or neurologists diagnose dementia following the Diagnostic and Statistical Manual of Mental Disorders, Fourth Edition. In clinics, patients suspected of dementia are assessed, and the possible cause is determined, based on medical history, daily life activities, physical condition, behavior, cognitive function, blood tests, and brain imaging. The end date was defined as the date on which dementia was diagnosed, loss to follow-up (e.g., death or withdrawal of insurance), or December 31^st^, 2010, whichever occurred first. A schematic description of the study design is presented in S1 Fig.

### Statistical analysis

Categorical variables are presented as numbers and percentages. Continuous variables are presented as mean ± standard deviation. For continuous unmatched data, differences among the three groups were assessed using the one-way ANOVA test, followed by Tukey’s *post hoc* test when appropriate. A Chi-squared test was used in similar comparisons for categorical data. In addition, for each PSM cohort, the standardized mean difference (SMD) was calculated as the difference between the means of the two groups divided by the pooled standard deviation. An absolute SMD value of less than 0.1 indicated a good balance. The incidence rate of dementia (per 1,000 person-year) for each group in the three CCs were also calculated. For PSM pairs, Cox proportional hazard models with a robust estimator (termed "robust Cox model" hereafter) [29] were adopted to obtain a precise estimation of the hazard ratio (HR) of developing dementia. The robust Cox model was adjusted for potential confounders such as annual ambulatory visit times, depression, peptic ulcer, and gastroesophageal reflux disease. Depression (ICD-9-CM code: 296.xx, 300.4, 311.xx) was identified at baseline period, while peptic ulcer (ICD-9-CM code: 531.xx-534.xx), GERD (ICD-9-CM code: 530.1, 530.8), and annual ambulatory visit times were identified during the follow-up period. Adjusted curves for cumulative risk of developing dementia between PSM pairs are also presented. Besides, the proportional hazard assumption was used to validate the application of robust Cox models. Since the risk of dementia is strongly associated with age, we further conducted subgroup analysis on different age groups (40–60 and > 60 years old). A two-tailed *P*-value < 0.05 was considered statistically significant. All analyses were performed using the SAS (version 9.2; SAS Institute Inc., Cary, NC, USA) and SPSS (version 19; IBM Corp., Armonk, NY, USA) software.

## Results

In total, 2,778 PPI users, 6,165 H2 antagonist users, and 86,238 non-users were included in this study. Table 1 presents the demographic characteristics of all three groups at baseline. Compared to non-users, PPI users were older and had a higher rate of comorbidities, except for depression. Similarly, compared to non-users, H2 antagonist users were also older and had a higher rate of comorbidities, except for chronic renal failure. Besides, compared to H2 antagonist users, PPI users were younger, had higher frequencies of diabetes mellitus, hyperlipidemia, and chronic renal failure, and lower frequencies of asthma and depression. The baseline demographic and clinical characteristics of the three CCs after PSM are shown in Table 2. For each CC, the absolute SMD value of baseline demographic and clinical characteristics was less than 0.1, indicating good balance after PSM. The mean follow-up duration of these CCs ranged between 4.0 and 4.4 years.

**Table 1:** Baseline characteristics of proton pump inhibitor, H2 antagonist, and non-user group before propensity score matching.

Baseline characteristics	PPI group	H2 antagonist group	Non-user group	*P*^d^
Numbers	2,778	6,165	86,238	
Age	56.0 ± 11.6^a^	56.9 ± 11.2^b^	53.4 ± 11.6^c^	< 0.01
Male	1,784 (64.2)^a^	2,972 (48.2)^b^	49,628 (57.6)^c^	< 0.01
Comorbidity				
Hypertension	664 (23.9)^a^	1,555 (25.2)^a^	11,877 (13.8)^b^	< 0.01
Diabetes mellitus	355 (12.8)^a^	633 (10.3)^b^	5,265 (6.1)^c^	< 0.01
CAD	227 (8.2)^a^	479 (7.8)^a^	3,476 (4.0)^b^	< 0.01
Hyperlipidemia	236 (8.5)^a^	418 (6.8)^b^	3,356 (3.9)^c^	< 0.01
Gout	148 (5.3)^a^	359 (5.8)^a^	2,582 (3.0)^b^	< 0.01
Stroke	78 (2.8)^a^	150 (2.4)^a^	1,633 (1.9)^b^	< 0.01
Asthma	76 (2.7)^a^	234 (3.8)^b^	1,260 (1.5)^c^	< 0.01
Chronic renal failure	41 (1.5)^a^	36 (0.6)^b^	402 (0.5)^b^	< 0.01
Depression	31 (1.1)^a^	115 (1.9)^b^	929 (1.1)^a^	< 0.01
Year of start date				
2002–2005	918 (33.0)	2,521 (40.9)	NA	< 0.01
2006–2009	1,860 (67.0)	3,644 (59.1)		

CAD, coronary artery disease; H2 antagonist, histamine-2 receptor antagonist; NA, not available; PPI, proton pump inhibitor

Data are shown as mean ± standard deviation for continuous variables or numbers (percentages) for categorical variables

NA due to no start date in non-user group before propensity score matching

^a-c^ Mean or percentage in a row without a common superscript letter differ (*P* < 0.05), as analyzed by one-way ANOVA test followed by Tukey’s *post hoc* test or Chi-squared test

^d^ Difference among three groups using one-way ANOVA test for continuous variables or Chi-squared test for categorical variables

**Table 2:** Characteristics of three comparison cohorts at baseline and during the follow-up period after propensity score matching.

PSM CC	CC1 (n = 2,583 pairs)			CC2 (n = 5,955 pairs)			CC3 (n = 2,765 pairs)		
Characteristics	PPI group	Non-user group	SMD	H2 antagonist group	Non-user group	SMD	PPI group	H2 antagonist group	SMD
Propensity score	0.19 ± 0.18	0.19 ± 0.18	< 0.01	0.08 ± 0.04	0.08 ± 0.04	< 0.01	0.33 ± 0.09	0.33 ± 0.09	< 0.01
Baseline									
Age	55.5 ± 11.5	55.8 ± 12.0	0.01	56.1 ± 11.1	56.9 ± 11.8	0.03	56.0 ± 11.6	55.7 ± 11.0	0.02
Male	1,647 (63.8)	1,653 (64.0)	< 0.01	2,873 (48.3)	2,866 (48.1)	< 0.01	1,773 (64.1)	1,772 (64.1)	< 0.01
Comorbidities									
Hypertension	597 (23.1)	560 (21.7)	0.03	1,465 (24.6)	1,510 (25.4)	0.02	657 (23.8)	647 (23.4)	0.01
Diabetes mellitus	305 (11.8)	285 (11.0)	0.02	609 (10.2)	599 (10.1)	0.01	345 (12.5)	328 (11.9)	0.02
Hyperlipidemia	210 (8.1)	218 (8.5)	0.01	392 (6.6)	350 (5.9)	0.03	228 (8.3)	228 (8.3)	0
CAD	203 (7.9)	169 (6.5)	0.05	457 (7.7)	386 (6.5)	0.05	223 (8.1)	231 (8.4)	0.01
Gout	128 (5.0)	122 (4.7)	0.01	331 (5.6)	294 (4.9)	0.03	147 (5.3)	150 (5.4)	< 0.01
Stroke	67 (2.6)	62 (2.4)	0.01	138 (5.6)	107 (1.8)	0.04	76 (2.8)	67 (2.4)	0.02
Asthma	68 (2.6)	63 (2.4)	0.01	215 (3.6)	179 (3.0)	0.03	76 (2.7)	67 (2.4)	0.02
Chronic renal failure	36 (1.4)	29 (1.1)	0.02	35 (0.6)	32 (0.5)	0.01	35 (1.3)	29 (1.1)	0.02
Depression	28 (1.1)	46 (1.8)	0.06	110 (1.9)	75 (1.3)	0.05	30 (1.1)	44 (1.6)	0.04
Year of the start date									
2002–2005	869 (33.6)	869 (33.6)	0	2,465 (41.4)	2,465 (41.4)	0	917 (33.2)	917 (33.2)	0
2006–2009	1,714 (66.4)	1,714 (66.4)	0	3,490 (58.6)	3,490 (58.6)	0	1,848 (66.8)	1,848 (66.8)	0
Follow-up									
Peptic ulcer	2,177 (84.3)	607 (23.5)	1.54	3,782 (63.5)	1,190 (20.0)	0.98	2,348 (84.9)	1,686 (61.0)	0.50
GERD	901 (34.9)	114 (4.4)	0.83	1,147 (19.3)	237 (4.0)	0.49	1,014 (36.7)	533 (19.2)	0.39
Annual ambulatory visit times	21.6 ± 21.0	16.5 ± 13.6	0.27	33.3 ± 25.5	12.3 ± 11.8	1.06	21.9 ± 21.2	33.6 ± 25.7	0.50
Follow-up period (years)	4.0 ± 2.0	4.0 ± 2.0	0.01	4.4 ± 2.2	4.4 ± 2.2	0.01	4.0 ± 2.0	4.0 ± 2.1	0.02

CAD, coronary artery disease; CC, comparison cohort; GERD, gastroesophageal reflux disease; H2 antagonist, histamine-2 receptor antagonist; PPI, proton pump inhibitor; PSM, propensity score matched; SMD, standardized mean difference

Data are shown as mean ± standard deviation for continuous variables or numbers (percentages) for categorical variables

Table 3 shows the incidence rate of dementia diagnosis and the results of multivariate robust Cox model analysis for each CC. After adjusting for confounding factors, the aHR (adjusted hazard ratio) of developing dementia was 0.72 [95% confidence interval (CI) = 0.51–1.03, *P* = 0.07)] in PPI users compared to the non-user group (CC1), which did not reach a significance level. For H2 antagonist users versus non-users (CC2), the association was also statistically insignificant (aHR = 0.95, 95% CI = 0.74–1.22, *P* = 0.69). Among acid suppressant users (CC3), the aHR of developing dementia in PPI users compared to H2 antagonist users was 0.82 (95% CI = 0.58–1.17, *P* = 0.28). In summary, no statistically significant differences were observed in any of the three CCs. S2–S4 Figs show adjusted curves for the cumulative risk of developing dementia in the three CCs. Further, we performed subgroup analysis by age group (40–60 and > 60 years old). Regardless of the age group, the results showed no association between the use of acid suppressants and dementia in each CC (S2 Table).

**Table 3:** Compare the risk of dementia in relation to acid suppressants use of three comparison cohorts after propensity score matching.

Comparison cohort	Dementia cases	Person-year	Incidence rate^a^ (95% CI)	Unadjusted HR (95% CI)	*P*	Adjusted HR^b^ (95% CI)	*P*
CC1 (n = 2,583 pairs)							
Non-user group	73	10,366.1	7.1 (5.6–8.9)	reference		reference	
PPI group	57	10,389.7	5.5 (4.2–7.1)	0.78 (0.55–1.10)	0.15	0.72 (0.51–1.03)	0.07
CC2 (n = 5,955 pairs)							
Non-user group	129	26,175.4	4.9 (4.1–5.9)	reference		reference	
H2 antagonist group	178	26,075.9	6.8 (5.9–7.9)	1.39 (1.11–1.74)	0.05	0.95 (0.74–1.22)	0.69
CC3 (n = 2,765 pairs)							
H2 antagonist group	78	10,966.8	7.1 (5.7–8.9)	reference		reference	
PPI group	62	11,058.2	5.6 (4.4–7.2)	0.79 (0.56–1.10)	0.16	0.82 (0.58–1.17)	0.28

CC, comparison cohort; CI, confidence interval; H2 antagonist, histamine-2 receptor antagonist; HR, hazard ratio; PPI, proton pump inhibitor

^a^ Incidence rate defined as dementia cases per 1,000 person-year

^b^ robust Cox proportional hazard model adjusted for annual ambulatory visit times, depression, peptic ulcer, and gastroesophageal reflux disease.

## Discussion

To date, the correlation between the use of acid suppressants and the risk of developing dementia remains controversial. In this study, we retrieved data from the same study population on PPI users, H2 antagonist users, and non-users and found no differences in the risk of developing dementia between any two groups after PSM. The exposure group was defined according to cDDD > 60 instead of ‘any use’ to ensure exposure to sufficient accumulating acid suppressant dosage. Besides, the start date of the follow-up period was defined as the date of cDDD > 60 in each of the acid suppressant groups to prevent immortal time bias, which is common in longitudinal studies [30, 31]. Furthermore, the PSM method was performed to minimize the possibility of confounding by indications [32].

We observed no association between the use of PPIs and the risk of developing dementia. This result is consistent with previous two population-based cohort studies, one conducted in the United States (402 PPI users, 3,082 non-users, aHR = 1.13, 95% CI = 0.82–1.56) [14] and the other in Korean, using the country’s claims database (1,947 PPI users, 68,086 non-users, aHR = 0.99, 95% CI = 0.70–1.39) [17]. In addition, a large-scale nested case-control study in Finland [16] [70,718 Alzheimer’s disease (AD) cases and 282,858 matched controls] and a case-control study in the UK [15] (25,811 AD cases and 25,811 matched controls) found no association between PPIs use and the risk of developing AD. Further support for our findings comes from reports that PPIs do not affect cognitive function decline [33, 34], which is a predictor of the risk of dementia development in the elderly [35]. In contrast, other studies have reported an increase in the risk of developing dementia following PPIs use [8–10]. Such discrepancies in findings might result from differences across studies in the definition for PPI exposure (e.g., cDDD of PPIs > 60 in our study versus any PPI use in the study by Tai et al. [10]). Conversely, several studies have implied that PPIs decrease the risk of developing dementia [13, 36]. These inconclusive findings may result from definition variations in the study population, methods of exposure assessment (e.g., self-reported [36] or database-retrieved [8–10, 13]), and/or drug exposure (e.g., cDDD of PPIs during the study period [10] or prescription of PPIs during every visit [8, 9, 36]).

In this study, we found no association between H2 antagonists use and the risk of developing dementia. This result is in agreement with two previous population-based cohort studies. One such study recruited 2,923 participants [19], while the other recruited 3,227 participants [37]. However, a case-control study (201 AD cases and 4,425 controls) has demonstrated that H2 antagonists reduce the risk of AD [20], and a longitudinal study (9,710 H2 antagonist users and 60,305 non-users) indicated that H2 antagonists increase the risk of developing dementia [17]. Possible reasons for these discrepancies include the difference in study design and/or drug exposure measurement (self-reported [37] or database-retrieved [17, 19]). Consequently, we used detailed information on drug names and dosage from the NHIRD claims database to define exposure and applied the PSM method to reduce the effects of confounding factors [38].

This study also compared the risk of developing dementia between PPI and H2 antagonist users. No association was observed here either. Previous research [21, 39], which used H2 antagonist users as a comparator group to evaluate the risk of developing dementia following PPIs use, had similar findings. H2 antagonists are used for similar indications as PPIs, reducing confounding by indication of the study [32]. However, the mechanisms of these two acid suppressants are different [4]. PPIs work by inhibiting the hydrogen/potassium adenosine triphosphatase signaling pathway in gastric parietal cells, thus leading to a reduced secretion of gastric acid into the gastric lumen. On the other hand, H2 antagonists decrease gastric acid secretion through competitive binding to histamine H2 receptors. In our study, while investigating the risk of developing dementia in acid suppressant users, we also compared PPI to H2 antagonist users for such a risk within the same population database. We found no difference between the groups in their respective risks. These findings could be helpful for physicians when appropriately prescribing acid suppressants, following clinical guidelines.

This study possesses several advantages. First, the PSM method was used to facilitate an impartial comparison between any two groups in our study design. Second, according to the rules of the Taiwan Food and Drug Administration, PPIs and H2 antagonists are classified as “prescription drugs” rather than “over-the-counter drugs.” Therefore, the NHIRD had complete nationwide coverage of these drugs. Third, the low rate of loss to follow-up (0.56%) minimized attrition bias. Nonetheless, there are also some limitations to this study. First, information on other potential influencing factors (e.g., the level of apolipoprotein E, lifestyle, education, and family history of dementia) was not available on the NHIRD. Also, NHIRD contains no information on early signs of dementia (e.g., immediate and delayed recall, forward digit span, and backward digit span) prior to diagnosis. Therefore, these risk factors or outcomes were not analyzed in this study. Second, a much longer follow-up period is desirable since the risk of developing dementia increases with age.

## Conclusion

According to the three CCs, using the population-based PSM cohort study, we found no association between acid suppressants use and the risk of developing dementia. These results help physicians to better prescribe acid suppressants in accordance with clinical guidelines. Further randomized controlled trials are warranted to confirm this relationship.

## Supporting information

S1 Table Anatomical therapeutic chemical code of proton pump inhibitors and histamine-2 receptor antagonists. (DOC)

S2 Table Subgroup analysis of adjusted hazard ratios of dementia in users of acid suppressants for three comparison cohorts. (DOC)

S1 Fig Scheme of study design for drug exposure group. (DOC)

S2 Fig Adjusted curves for the cumulative risk of developing dementia in PPI group and non-user group (comparison cohort 1). (DOC)

S3 Fig Adjusted curves for the cumulative risk of developing dementia in H2 antagonist group and non-user group (comparison cohort 2). (DOC)

S4 Fig Adjusted curves for the cumulative risk of developing dementia in PPI group and H2 antagonist group (comparison cohort 3). (DOC)
