# Correction: Acid suppressants use and the risk of dementia: A population-based propensity score-matched cohort study

**DOI:** 10.1371/journal.pone.0250535

**Published:** 2021-04-15

**Authors:** The PLOS ONE staff

There is an error in affiliation 1 for authors Chia-Liang Wu and Jaw-Shing Wang. The publisher apologizes for the error. The correct affiliation 1 is: Department of Psychiatry, Taipei Veterans General Hospital, Yuli Branch, Hualien City, Taiwan.
