# Supplementary material for: Acid suppressants use and the risk of dementia: A population-based propensity score-matched cohort study
Source: PLoS One. 2020 Nov 30;15(11):e0242975. doi: 10.1371/journal.pone.0242975 (PMC7703973; doi:10.1371/journal.pone.0242975)
Supplement: S1 Table — (DOC) [file pone.0242975.s001.doc]

**S1 Table. Anatomical Therapeutic Chemical code of proton pump inhibitors and histamine-2 receptor antagonists**

| Medications | Anatomical Therapeutic Chemical code |
| --- | --- |
| Proton pump inhibitors |  |
| Omeprazole | A02BC01 |
| Pantoprazole | A02BC02 |
| Lansoprazole | A02BC03 |
| Rabeprazole | A02BC04 |
| Esomeprazole | A02BC05 |
| Dexlansoprazole | A02BC06 |
| Dexrabeprazole | A02BC07 |
| Histamine-2 receptor antagonists |  |
| Cimetidine | A02BA01 |
| Ranitidine | A02BA02 |
| Famotidine | A02BA03 |
| Nizatidine | A02BA04 |
| Niperotidine | A02BA05 |
| Roxatidine | A02BA06 |
| Ranitidine bismuth citrate | A02BA07 |
| Lafutidine | A02BA08 |
