# Supplementary material for: Acid suppressants use and the risk of dementia: A population-based propensity score-matched cohort study
Source: PLoS One. 2020 Nov 30;15(11):e0242975. doi: 10.1371/journal.pone.0242975 (PMC7703973; doi:10.1371/journal.pone.0242975)
Supplement: S2 Table — (DOC) [file pone.0242975.s002.doc]

**S2 Table. Subgroup analysis of adjusted hazard ratios of dementia in users of acid suppressants for three comparison cohorts**

| Age group  (years) | CC1 |  |  | CC2 |  |  | CC3 |  |  |
| --- | --- | --- | --- | --- | --- | --- | --- | --- | --- |
|  | N | aHR^a^ (95 % CI) | *P* | N | aHR^a^ (95 % CI) | *P* | N | aHR^a^ (95 % CI) | *P* |
| 40 – 60 | 3,433 | 0.67 (0.26, 1.74) | 0.41 | 7,372 | 1.09 (0.53, 2.21) | 0.82 | 3,582 | 1.27 (0.45,3.62) | 0.65 |
| > 60 | 1,733 | 0.73 (0.50, 1.07) | 0.11 | 4,538 | 0.93 (0.71, 1.20) | 0.56 | 1,948 | 0.82 (0.56,1.19) | 0.30 |

aHR, adjusted hazard ratio; CC, comparison cohort; CI, confidence interval. CC1: PPI users compared to a non-users group, CC2: H2 antagonist users compared to a non-users group, and CC3: PPI users compared to H2 antagonist users group.

^a^ Adjusted for annual ambulatory visit times, depression, peptic ulcer, and gastroesophageal reflux disease
