# Supplementary material for: Acid suppressants use and the risk of dementia: A population-based propensity score-matched cohort study
Source: PLoS One. 2020 Nov 30;15(11):e0242975. doi: 10.1371/journal.pone.0242975 (PMC7703973; doi:10.1371/journal.pone.0242975)
Supplement: S1 Fig — (DOC) [file pone.0242975.s003.doc]

S1 Fig. Scheme of study design for drug exposure group

HIV, human immunodeficiency virus

Indicates drug prescription

Study period: 2001 to end date

Follow-up period: Start to end dates

Start date: The date of drug prescription > 60 cumulative defined daily dose during the study period

End date: The date of dementia diagnosis, loss of follow-up (e.g., death or withdrawal of insurance), or December 31^st^, 2010, whichever occurred first.
