# Supplementary material for: Acid suppressants use and the risk of dementia: A population-based propensity score-matched cohort study
Source: PLoS One. 2020 Nov 30;15(11):e0242975. doi: 10.1371/journal.pone.0242975 (PMC7703973; doi:10.1371/journal.pone.0242975)
Supplement: S3 Fig — (DOC) [file pone.0242975.s005.doc]

HR (95% CI) = 0.95 (0.74-1.22)

*P* = 0.69

| **H2 antagonist** | 5955 | 5955 | 4897 | 3934 | 3096 | 2374 | 1649 | 874 | 371 |
| --- | --- | --- | --- | --- | --- | --- | --- | --- | --- |
| **Non-user** | 5955 | 5955 | 4892 | 3939 | 3110 | 2388 | 1677 | 898 | 380 |
| **Number at risk** | | | | | | | | | |

**S3 Fig. Adjusted curves for the cumulative risk of developing dementia in H2 antagonist group and non-users group (comparison cohort 2).** A representation of the adjusted cumulative risk of dementia for propensity score matching samples based on robust Cox model. Covariates included annual ambulatory visit times, depression, peptic ulcer, and gastroesophageal reflux disease. Subjects with a follow-up period of less than one year were excluded. CI, confidence interval; H2 antagonist, histamine-2 receptor antagonist; HR, hazard ratio
